# DWI scalp dot sign: superficial temporal artery restricted diffusion in giant cell arteritis

**DOI:** 10.1093/rheumatology/keac502

**Published:** 2022-09-01

**Authors:** Eleanor Taylor, Francesca Tona, Victoria Singh-Curry, Marius Venter, Maresa Carulli, Colin Tench, Taryn Youngstein, Luke Dixon

**Affiliations:** Neuroradiology Imaging Department, Charing Cross Hospital, Imperial College Healthcare NHS Trust, London, UK; Neuroradiology Imaging Department, Charing Cross Hospital, Imperial College Healthcare NHS Trust, London, UK; Neuroradiology Imaging Department, Charing Cross Hospital, Imperial College Healthcare NHS Trust, London, UK; Neuroradiology Imaging Department, Charing Cross Hospital, Imperial College Healthcare NHS Trust, London, UK; Neuroradiology Imaging Department, Charing Cross Hospital, Imperial College Healthcare NHS Trust, London, UK; Neuroradiology Imaging Department, Charing Cross Hospital, Imperial College Healthcare NHS Trust, London, UK; Neuroradiology Imaging Department, Charing Cross Hospital, Imperial College Healthcare NHS Trust, London, UK; Neuroradiology Imaging Department, Charing Cross Hospital, Imperial College Healthcare NHS Trust, London, UK

Rheumatology key message

- Restricted diffusion in the superficial temporal arteries may serve as a further radiological sign of giant cell arteritis.

Dear Editor, We would like to report a novel finding on standard diffusion-weighted magnetic resonance imaging (DWI-MRI) in a case series of four patients with giant cell arteritis (GCA) in the superficial temporal artery (STA). GCA is considered a clinical emergency and is the most common vasculitis in adults, which, if untreated, can rapidly cause irreversible sight loss and ischaemic stroke [1]. Unfortunately, clinical presentation is variable and classic symptoms such as headache and scalp tenderness can be absent, delaying diagnosis and therapy [1]. Temporal artery biopsy (TAB) is considered the reference standard for diagnosis, but sensitivity can be as low as 40% due to skip lesions and inadequate specimens [2]. The European Alliance of Associations for Rheumatology recommends temporal and axillary artery ultrasound (US) as a first-line investigation in patients suspected of having GCA [3]. The finding of an arterial halo on US, reflecting inflammatory oedema of the arterial wall, is thought diagnostic for GCA with a specificity of 96% [4]. However, the sensitivity of US is lower (43–77%) and is highly technique and operator dependent [4, 5]. In both TAB and US, sensitivity drops further if performed after commencing steroid therapy. Post-contrast vessel wall magnetic resonance imaging (VW-MRI) has been proposed as a potential alternative first-line imaging investigation, with experienced units reporting a sensitivity of 80% and a specificity of 100% [5]. VW-MRI is still a relatively new technique and not yet readily available.

We report a novel finding on routine standard DWI-MRI of restricted diffusion in the STAs in four patients with subsequently confirmed GCA.

The imaging of four patients with a subsequent diagnosis of GCA, who had an initial routine MRI head on a 3 T (case 1, 2) or 1.5 T (case 3, 4) scanner for varied acute neurological presentations, was retrospectively reviewed. Informed consent was obtained for all participants. They were male with an age range between 65 and 90 years. Clinical presentations included: bilateral frontal headache and diplopia (case 1, for 3 weeks), dysarthria and exotropia (case 2, for 8–10 weeks), right vision impairment (case 3, for 24 h) and right temporal headache and transient right upper limb weakness (case 4, for 10 days). CRP ranged between 7 and 173 mg/l, and ESR was elevated in all patients (50–87 mm/h). The final diagnosis of GCA was based on either US (cases 2 and 3) or TAB (cases 1 and 4). In case 1, US was inconclusive necessitating TAB. In case 4, US was not performed. In all four cases, MRI of the head revealed abnormal restricted diffusion in the STA branches on high *b*-value (*b* = 1000) DWI images, appearing as a ‘dot’ of diffusion restriction at the level of the scalp, in the anatomical region of the STA branches on axial sequences (see Fig. 1). In the cases where dedicated VW-MRI was also performed (cases 1 and 2), the regions of apparent arterial restricted diffusion also exhibited abnormal vessel wall enhancement. The distribution of arterial DWI abnormality correlated with the US findings in all three cases where US was performed. Resolution of restricted diffusion was demonstrated in all three cases where a follow-up MRI was performed following steroid therapy. Detailed imaging and clinical information are provided in Supplementary Table S1, available at *Rheumatology* online.

**Figure 1.**
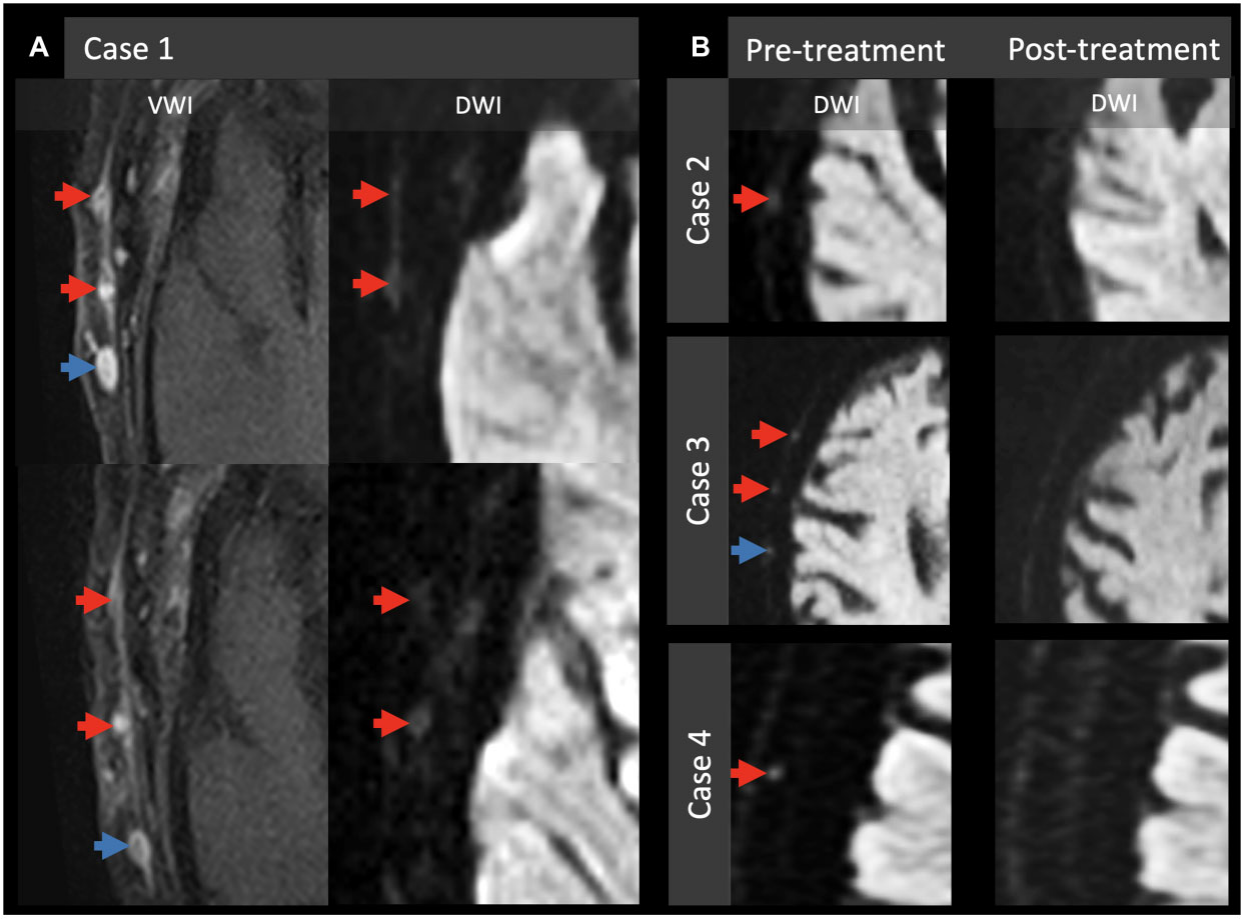
Four patients with GCA all demonstrating restricted diffusion related to the superficial temporal arteries. (**A**) Left, MRI of case 1 at presentation showing concentric wall thickening and enhancement of the right STA branches on VWI. Right, DWI of the same regions demonstrating high DWI signal and low apparent diffusion coefficient (not shown) in the same STA branches. (**B**) Three further cases where restricted diffusion is demonstrated in the STA branches (left column), which resolved on the post-therapy MRI (right column). Red arrows, STAs; blue arrows, superficial temporal veins. DWI: diffusion-weighted imaging; STA: superficial temporal artery; VWI: vessel wall imaging

DWI is an MRI technique routinely included in all standard MRI head protocols, generating images upon measuring the random Brownian motion of water molecules within the tissues. Cellular infiltration associated with acute inflammation restricts the movement of water molecules resulting in high DWI and low apparent diffusion coefficient signal. The ability of DWI to detect tissue inflammation is well established, but its role in diagnosing superficial temporal arteritis has not been explored [6]. We postulate that the diffusion restriction noted in the STA branches reflects active vessel wall inflammation, which is supported by the observed resolution of diffusion restriction following steroid therapy. This finding is mirrored by preliminary studies looking at specialized whole-body DWI in extracranial large-vessel GCA [7], which found mural restricted diffusion in the aorta and axillary arteries of eight patients with GCA, which correlated with disease activity on fluorodeoxyglucose (FDG)-PET/CT. Our series is the first to note STA restricted diffusion on standard conventional DWI head imaging. The greatest challenge for a confident diagnosis of GCA is early access to diagnostic testing as mural inflammation resolves rapidly with steroids. Unfortunately, US, FDG-PET/CT and TAB are often not rapidly available and vessel wall imaging-MRI, whilst very promising, is a relatively new technique that is not offered at many hospitals. Despite this being only a small case series, these preliminary findings suggest that DWI-MRI is a potential, already available, fast, non-invasive tool in the diagnosis of GCA in the STA. Further investigation with prospective studies assessing the diagnostic accuracy of ‘DWI scalp dot sign’ is needed.

## Supplementary data

Supplementary data are available at *Rheumatology* online.

## Supplementary Material

keac502_Supplementary_Data

## Data Availability

Data are available upon reasonable request by any qualified researchers who engage in rigorous, independent scientific research, and will be provided following review and approval of a research proposal and Statistical Analysis Plan (SAP) and execution of a Data Sharing Agreement (DSA). All data relevant to the study are included in the article.

## References

[1] van der Geest KSM , Sandovici M, Brouwer E et al Diagnostic accuracy of symptoms, physical signs, and laboratory tests for giant cell arteritis. JAMA Intern Med 2020;180:1295. 32804186 10.1001/jamainternmed.2020.3050 PMC7432275

[2] Luqmani R , Lee E, Singh S et al The role of ultrasound compared to biopsy of temporal arteries in the diagnosis and treatment of giant cell arteritis (TABUL): a diagnostic accuracy and cost-effectiveness study. Health Technol Assess (Rockv) 2016;20:1–238. 10.3310/hta20900 PMC5165283 27925577

[3] Dejaco C , Ramiro S, Duftner C et al EULAR recommendations for the use of imaging in large vessel vasculitis in clinical practice. Ann Rheum Dis 2018;77:636–43. 29358285 10.1136/annrheumdis-2017-212649

[4] Arida A , Kyprianou M, Kanakis M et al The diagnostic value of ultrasonography-derived edema of the temporal artery wall in giant cell arteritis: a second meta-analysis. BMC Musculoskelet Disord 2010;11:44. 20210989 10.1186/1471-2474-11-44 PMC2837862

[5] Poillon G , Collin A, Benhamou Y et al Increased diagnostic accuracy of giant cell arteritis using three-dimensional fat-saturated contrast-enhanced vessel-wall magnetic resonance imaging at 3 T. Eur Radiol 2020;30:1866–75. 31811430 10.1007/s00330-019-06536-7

[6] Gasparetto EL , Cabral RF, da Cruz LC, Domingues RC. Diffusion imaging in brain infections. Neuroimaging Clin N Am 2011;21:89–113, viii. 21477753 10.1016/j.nic.2011.01.011

[7] Ironi G , Tombetti E, Napolitano A et al Diffusion-weighted magnetic resonance imaging detects vessel wall inflammation in patients with giant cell arteritis. JACC Cardiovasc Imaging 2018;11:1879–82. 30121275 10.1016/j.jcmg.2018.06.015

