# Supplementary material for: DWI scalp dot sign: superficial temporal artery restricted diffusion in giant cell arteritis
Source: Rheumatology (Oxford). 2022 Sep 1;62(4):e119–21. doi: 10.1093/rheumatology/keac502 (PMC10070058; doi:10.1093/rheumatology/keac502)
Supplement: keac502_Supplementary_Data [file keac502_supplementary_data.docx]

Supplementary Table S1

| **Case** | **Ultrasound of superficial temporal artery** | **Distribution of restricted diffusion** | **Patchy or diffuse restricted diffusion** | **TAB** | **Time of MR follow up** | **Treatment** |
| --- | --- | --- | --- | --- | --- | --- |
| **1** | No halo sign but diffuse mural thickening in bilateral common STA, parietal and frontal branches. | Right common STA, bilateral frontal and parietal branches | Diffuse | Positive | 3 weeks | IV methylprednisolone 500mg for 1 day, than 1gr for 2 days, than 40mg oral prednisolone OD for 3/52 then 30mg for 3/52 then 20mg for 3/52 then 10 mg for 3/52 |
| **2** | Halo sign in common STA, frontal and parietal branches bilaterally. | Bilateral common STA, frontal and parietal branches. | Diffuse | Not performed | 1 month | Oral prednisolone OD 50mg for 3/52 then 40mg for 2/52 then 30mg for 2/52 then 20mg for 4/52 then reduce by  2.5mg monthly until 10mg. |
| **3** | Halo sign bilateral frontal branches. | Bilateral common STA, bilateral frontal, and left parietal branches. | Diffuse | Not performed | 1 year | IV methylprednisolone 500mg for 3 days then 60 mg oral prednisolone OD for 3/52 then 50 mg for 3/52 then 40mg for 3/52 then 30mg for 3/52 then 20mg for 3/52 then 10 mg for 3/52 |
| **4** | Not performed. | Right frontal and parietal branches | Patchy | Positive | 4 months | Oral prednisolone 40mg OD  for 3/52 than 30 mg for 2/52 than 25mg for 2/52 than 20 mg for 2/52  Then reduce by 2.5 mg monthly until 10 mg. |
